# Whole-genome sequence of subcluster BE1 *Streptomyces lividans* bacteriophage Persimmon

**DOI:** 10.1128/mra.00024-25

**Published:** 2025-04-29

**Authors:** Wanji Li, Meiru Shang, Kathleen Weston Hafer, Christopher D. Shaffer

**Affiliations:** 1 Department of Biology, Washington University in St Louis 123752, St. Louis, Missouri, USA; Portland State University, Portland, Oregon, USA

**Keywords:** bacteriophage, streptomyces, siphovirus, genome

## Abstract

Persimmon is a BE1 subcluster bacteriophage infecting *Streptomyces lividans* with siphoviral morphology and was isolated from a soil sample. The genome of Persimmon has a length of 131,421 bp, 231 protein-coding genes, and a 50.0% GC content that differs from the isolation host with high GC content.

## ANNOUNCEMENT

*Streptomyces* is of special interest in biotechnology for its potential in developing protein secretion and cell-free expression systems (1, 2). Identifying *Streptomyces* bacteriophages could facilitate these applications by providing biotechnological tools derived from bacteriophages (3, 4). Here, we report the genome sequence of Persimmon, a BE1 subcluster bacteriophage infecting *Streptomyces lividans* (5).

**Fig 1:**
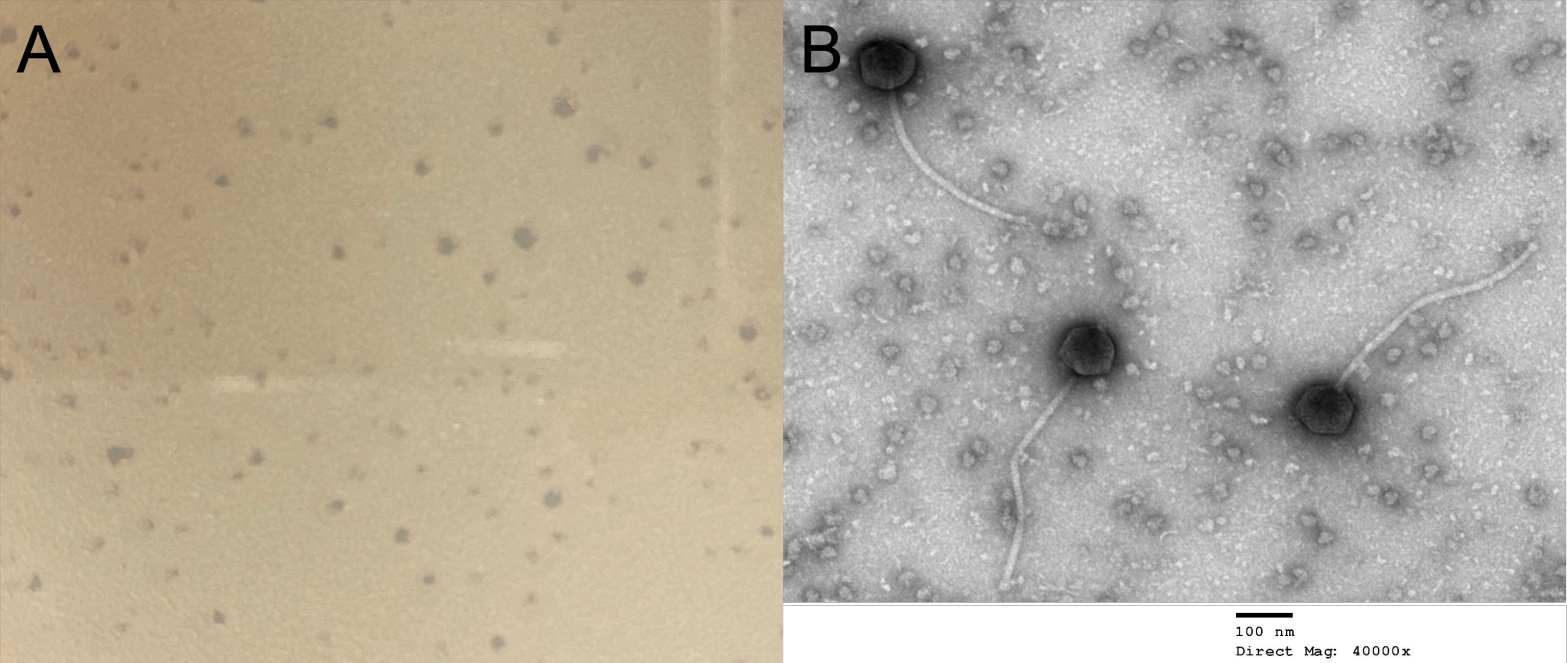
(**A**) Clear and round plaques (about 1 mm in diameter) formed by Persimmon on *Streptomyces lividans* top agar plate. (**B**) Transmission electron microscopy (TEM) of Persimmon, with an average tail length, head length, and head width of 349 nm, 78 nm, and 73 nm, respectively (*n* = 39). Scale bar is 100 nm.

Persimmon was directly isolated from a soil sample (GPS 38.643333 °N, 90.291389 °W) at a depth of 8 cm on 6 September 2021 (6). Briefly, the soil sample was washed in Difco nutrient broth supplemented with 8 mM Ca(NO_3_)_2_ and 0.5% (w/v) glucose. The filtered broth (0.02 µm pore size) was plated, covered with top agar containing about 10^6^ viable spores (NRRL B-16148), and incubated at 30°C. Following three rounds of purification, Persimmon formed clear, round plaques with a diameter of about 1 mm (Fig. 1A). TEM by negative stain (1% uranyl acetate) revealed a siphoviral morphology (Fig. 1B). Tail length, head length, and head width were measured by Fiji (7), which are 349 ± 16 nm, 78 ± 5 nm, and 73 ± 3 nm, respectively (*n* = 39, mean ± standard deviation).

Persimmon genomic DNA was extracted from a lysate with the DNeasy Blood & Tissue Kit (QIAGEN, Cat. No. 69506). A Kapa Biosystems high-throughput library was prepared and sequenced on Illumina NovaSeq 6000 with dual index 2 × 150 reads. The reads were trimmed by Trimmomatic version 0.38 (8) and assembled by Newbler (Roche). The final 154× coverage assembly was verified by manual inspection using Consed v29.0 (9). The genome is 131,421 bp long and has 50.0% GC content, with a direct terminal repeat of 10,610 bp as identified by approximately twofold increase in read coverage. Persimmon was assigned to the BE1 subcluster based on gene content similarity (10). The results are available in the Actinobacteriophage database (11).

Positional annotations were automatically assigned using DNA Master v5.23.6 (http://cobamide2.bio.pitt.edu/), Glimmer v3.02 (12), GeneMark v2.5p (13), Starterator v1.2 (https://github.com/SEA-PHAGES/starterator), NCBI BLAST searching nonredundant and Actinobacteriophage databases (14, 15), and Phamerator (16). Functional annotations were made by HHpred searching PDB mmCIF70, Pfam-A, and SCOPe databases (17, 18), BLASTp (14), and DeepTMHMM (19, 20). tRNAs and tmRNAs were annotated using ARAGON v1.2 (21) and tRNAscan-SE v2.0 (22). All software was used with default settings. Finally, 231 protein-coding genes, 45 tRNAs, and 1 tmRNA were identified.

The GC content of the Persimmon genome deviates from its host (50.0% versus 72.2%) but aligns with other BE1 subcluster bacteriophages (23, 24). A similar observation was discovered in mycobacteriophage Patience (25). Potential deleterious effects derived from the bacteriophage-host GC content disparity might be ameliorated by Persimmon’s large tRNA and tmRNA repertoire.

The 231 genes in Persimmon represent 208 protein families (phams), among which 96 are exclusive to cluster BE bacteriophages and predominantly encode enzymes and nucleic acid-binding proteins. The remaining 112 phams primarily consist of structural and assembly genes. Additionally, the absence of integrase or repressor genes in cluster BE phages suggests a lytic lifestyle.

## Data Availability

Persimmon is available at GenBank under accession no. OR253896 and Sequence Read Archive (SRA) no. SRX25999125.
